# Short time effect of a self-referral to inpatient treatment for patients with severe mental disorders: a randomized controlled trial

**DOI:** 10.1186/s12913-016-1712-z

**Published:** 2016-09-22

**Authors:** Inger Elise Opheim Moljord, Kristel Antine Helland-Hansen, Øyvind Salvesen, Turid Møller Olsø, Camilla Buch Gudde, Marit By Rise, Aslak Steinsbekk, Lasse Eriksen

**Affiliations:** 1 Division of Psychiatry, Nidaros Community Mental Health Center, St. Olav’s University Hospital, Østmarkveien 21, Post box 1893 Lade, N-7440 Trondheim, Norway; 2 Department of Neuroscience, Faculty of Medicine, Norwegian University of Science and Technology, Trondheim, Norway; 3 Division of Psychiatry, Department of Research and Development, St. Olav’s University Hospital, Trondheim, Norway; 4 Department of Public Health and General Practice, Faculty of Medicine, Norwegian University of Science and Technology, Trondheim, Norway; 5 Norwegian Resource Center for Community Mental Health, Norwegian University of Science and Technology Social Research AS, Trondheim, Norway; 6 Forensic Department Brøset, Center for Research and Education in Forensic Psychiatry, St. Olav’s University Hospital, Trondheim, Norway; 7 Department of Social Work and Health Science, Faculty of Social Science and Technology Management, Norwegian University of Science and Technology, Trondheim, Norway

**Keywords:** Self-referral to inpatient treatment, Patient controlled admission, Psychosis, User participation, Patient activation

## Abstract

**Background:**

Service user participation is a central principle in mental healthcare, and the opportunity to self-refer to inpatient treatment is used to increase service user involvement and activation. The aim of this study was to investigate the short-term effect of a self-referral system in an inpatient rehabilitation unit at a community mental health center on patient activation and recovery in individuals with severe mental disorders.

**Methods:**

A randomized controlled study including 53 patients (41 % females, mean age 40 years). Twenty-six patients in the intervention group were given a contract for self-referral to inpatient treatment, limited to maximum 5 days and a quarantine time of 14 days between each stay. The control group (27 participants) received treatment as usual, and was offered the intervention after 1 year. The Patient Activation Measure was the primary outcome and secondary outcome was the Recovery Assessment Scale. Mixed models were used to assess group differences.

**Results:**

During the 4 months period, 15 (58 %) of 26 participants in the intervention group used the contract of self-referral to inpatient treatment. The intervention group had more admissions than the control group but both groups had a similar total use of inpatient days and out-patient consultations. The self-referral to inpatient treatment counted for 11 % of all inpatient days for the intervention group. There were no significant differences in the outcome between the groups on patient activation (estimated mean difference 2.7, 95 % confidence interval = −5.5 to 10.8, *p =* 0.52) or recovery (estimated mean difference 0.01, 95 % confidence interval = −0.3 to 0.3, *p* = 0.92).

**Conclusions:**

Giving persons with severe mental disorders the possibility to self-refer to inpatient treatment did not change their level of patient activation and recovery after 4 months and did not lead to increased use of health services. The cost-effectiveness and long-term effect of self-referral to inpatient treatment should be investigated further.

**Trial registration:**

NCT01133587, clinicaltrials.gov.

## Background

Service user participation is a central principle in mental healthcare [1]. Research suggests that service users wish to be involved in their treatment decisions [2, 3] and to play an active part [3–5]. To improve service user participation an increasing number of care models have been highlighted in mental health policy documents [6]. Such models include building relationship through participation and efforts to increase the independence of persons with mental health problems [7, 8]. The care model in mental health should be balanced between hospital and community care and its service system [9]. Many people with severe mental disorders need periodical timely inpatient treatment to avoid adverse events, worsening of symptoms and prolonged hospital stays. It is reported that even small changes in signs and symptoms can predict future disorders up to 10 weeks in advance [10]. Recognizing early symptoms can be difficult, and together with waiting time for treatment this might result in increased admissions [10, 11]. Thus, it is important to implement a model for early warning training and relevant patient actions as well as the possibility to implement these actions to reduce risk of relapse [10].

One way to combine more timely treatment with increased user participation is to open for self-referral to inpatient treatment (SRIT). SRIT is a model that involves the patients to take a more active role in treatment decisions, admission process, and should stimulate increase knowledge of disorder and identification of early signs, and thereby result in reduction of hospital stay. In a recent systematic review based on qualitative and observational studies of SRIT, it was suggested that self-referral supports early help seeking, increases patient autonomy and coping, avoids power struggles, reduces the total time of hospitalization, and prevents compulsory admissions [12]. A new qualitative article nested from the same project as the present study indicated that the participant found it important to have the option to decide admission for oneself and to have services that focus on individual needs [13]. This seems to improve service users’ confidence, both in their services and their own skill to cope with everyday life [13]. One of the aims of SRIT is to increase service user participation and thereby patient activation. High patient activation is associated with improved hope [5], and optimistic attitudes to recovery [5], better coping strategies, decreased mental health symptoms [14], positive self-management [15, 16] and reduced substance use disorders [16]. One of the central principles of recovery is that the service users can build and live a meaningful, satisfying, and hopeful life defined by themselves through taking more control over their lives in spite of their disorders [17]. These goals all require health service organizations to be more oriented towards recovery [8].

Recently, the first systematic review of this service model identified six studies from four different research projects, all with low levels of evidence [12], according to the Oxford Centre for Evidence-Based Medicine classification [18]. Since no randomized controlled trial (RCT) has so far been published, the promise of SRIT as a model in mental healthcare has limited basis for firm conclusions [12].

The aim of the present study was to investigate the effect of a contract for SRIT on patient activation and recovery in patients with severe mental disorders after 4 months, compared with treatment as usual (TAU) in a randomized, controlled study. As our primary hypothesis we expected a significant improvement in patient activation from baseline to 4 months in the SRIT group, compared to the TAU group. The secondary hypothesis was significant improvement in recovery from baseline to 4 months in the SRIT group, compared to the TAU group.

## Methods

### Trial design

We conducted an open parallel group randomized controlled trial (RCT). The trial was registered in clinicaltrials.gov (NCT01133587).

The study took place at a community mental health center (CMHC) in Central Norway. The inclusion period was between May 2010 and December 2012.

In order to have closer monitoring of participants during the project, the first follow up was moved forward from the planned 6 months to 4 months. The change was implemented after the first participant had finished the 6 months assessment but before any other participants had been in the study for more than 4 months (i.e., for one participant the 6-month outcomes were used).

### Participants

The inclusion criteria were adult patients with severe mental disorders. All participants, except for two, were clinically diagnosed with bipolar disorders or schizophrenia. One participant was diagnosed with an organic psychosis and one with a personality disorder with psychotic features (voice hallucinations). Some patients had several diagnoses. The participants were well known at the CMHC rehabilitation unit and in need of continued long-term care from both primary and specialist healthcare. All patients had to be known at the unit before randomization. Exclusion criteria were patients with severe substance abuse problems or self-destructive behavior, and those unable to consent or deemed unable to use SRIT as intended. An interdisciplinary team decided who was eligible as part of the department’s treatment meetings.

The recruitment took place by informing patients and staff at the CMHC both orally and in writing. The participants volunteered themselves or were recommended by their therapists. All participants had to be approved for the study by the chief physician. Some of the patients were included and randomized while they were still admitted at the CMHC, based on an expectation that they would be discharged within a few days.

### Intervention

The purpose of a SRIT contract was to increase user participation and to offer patients with increased symptoms easy access to inpatient treatment without contacting the doctor in advance. Additionally, the SRIT intervention was meant to increase the responsibility for one’s own health, which includes supporting and empowering SRIT patients to make their own decisions considering use of the contract.

Before randomization all participants and staff received information about SRIT and its routines prior to inclusion, and that two beds were completely reserved for the SRIT project. All participants were informed that a SRIT contact could be used when they needed it (e.g. “a time out”, structure during the day, experienced warning signs and increased mental health symptoms). There was a focus on having treatment in time to avoid increased symptoms of mental disorders. They were also informed that a SRIT contract implied in the worst case a few days waiting time for a vacant bed. All participants were motivated to establish an individual plan, which all patients with severe mental disorders have a right to have [19, 20]. Almost all patients had an individual plan when they joined the study.

After randomization the SRIT participants went through the SRIT contract with the researcher and staff. The participants went through their individually warning signs and what they could do for reducing those symptoms, together with their therapist.

The SRIT participants could contact the staff or researchers directly for supplementary information. Participants assigned to the SRIT intervention could self-refer to the rehabilitation section at the CMHC on Mondays to Fridays between 08:00 and 20:00. If they needed to stay over the weekend, they had to contact the unit before 15:30 on Friday. They could stay for 5 days, with a minimum of 14 days quarantine between each stay to avoid capacity problems in the unit. This was based on suggestions from the first study on self-referral in Norway [21]. Participants followed the usual rules and structure of the unit, such as meal times and activities of daily living. When they self-referred, they had a consultation with a specialist nurse in psychiatry who documented the basis of the consultation in the health record. Normally, there were no changes to medication during the stay but if needed an appointment with a psychiatrist was arranged. All patients could have ordinary admissions at the CMHC or hospitals by a doctor via normal procedures.

Participants randomized to TAU were informed that they would receive a SRIT contract after 1 year if they still satisfied the inclusion criteria. They followed ordinary procedures, contacting their general practitioner, emergency department, or duty doctor if they needed hospitalization. They could not call the CMHC directly to ask for treatment.

To describe the implementation of the intervention, data were taken from the Patient Administrative System of the hospital. The researchers had no influence on this system. The use of SRIT could lead to a change in use of other services, both the use of the SRIT and use of other health care services at the hospital were recorded.

### Outcome measurement

The questionnaires were completed at baseline and after 4 months. Some of the participants preferred to answer the follow up questionnaires in their own home, but most got an appointment with the researchers and completed them at the CMHC. A few asked for help in understanding the questions.

The primary outcome was patient activation measured using the Patient Activation Measure (PAM) [22, 23]. This is a 13-item self-report scale, measuring patient knowledge, skill, and confidence in self-management. It is scored on a five-point Likert scale as follows: 0 = not applicable; 1 = strongly disagree; 2 = disagree; 3 = agree; and 4 = strongly agree [22, 23]. The sum of raw scores from the PAM was transformed to a 0–100 scale where higher scores indicated higher patient activation [23, 24]. The transformation is based on the new index-scale by Insignia [24]. PAM has been validated among several patient groups with various chronic diseases [15, 25–27]. In this study, we used the Norwegian back-translated PAM [28], which has been validated in mental health populations [29].

The secondary outcome was recovery measured using the Recovery Assessment Scale (RAS) [30]. This 24-item scale, is scored on a five point Likert scale (1 = strongly disagree, 5 = strongly agree). A mean subscale score was calculated. RAS is designed to measure recovery from severe mental disorders [30] and has been found to be appropriate for measuring recovery [31]. The scale measures personal confidence and hope, willingness to ask for help, goal and success orientation, reliance on others, and degree of domination by symptoms. The RAS has shown good test-retest reliability, as well as good internal consistency [30, 32–35]. The scale was translated to Norwegian for this study using forward and back translation with two independent persons doing each of the translations.

### Sample size

A sample size calculation was conducted using a two-sample *t*-test to find a difference in PAM scores between the groups of 10. With an equal standard deviation of 11, significance level of 0.05 and 80 % power, 21 people in each arm was needed. To allow for drop-outs it was aimed for 60 participants, consistent with the recommendation of Rosenthal and Rosnow to add one third of the minimum number according to the power analysis to compensate for possible drop-out [36].

### Randomization

Block randomization was performed using a web-based randomization system (WebCRF, version 1.3) developed and administered by the Unit of Applied Clinical Research, Institute of Cancer Research and Molecular Medicine, Norwegian University of Science and Technology, Trondheim, Norway. The randomization was balanced 1:1, and stratification was done for whether or not patients were using a special outpatient follow-up service (Psychiatric Ambulatory Rehabilitation Team) which was assumed to provide extra support for their users.

### Blinding

There was no blinding in the study. The statistician who analysed the data was blinded to group allocation.

### Statistical methods

Patient characteristics and description of the implementation in terms of use of health services for the two groups were compared using the independent samples *t*-test for continuous variables, Chi-square for categorical variables, and the Mann–Whitney U for non-parametric continuous variables, all two-tailed. The distributions of PAM and RAS scores were examined and found to be approximately normally distributed.

The effect of the intervention was assessed using intention to treat (ITT) and per protocol procedures.

Those who could not use the contract (e.g. moved, died, long-term hospitalization) were not included in the per protocol analysis. This turned out to be three persons in the SRIT group, two were long termed treated in hospital and one did not want to have further contact with the CMHC. As the per protocol analyses gave the same results as the ITT analysis, the results are not shown.

The intention to treat analysis [37] was done using a linear mixed model which uses all available data in the presence of dropouts and there is no need for multiple imputations [38]. To account for within-subject correlation, patient identification was specified as a random effect. The effect of the intervention and time were specified as a fixed effect with the following three levels: (1) baseline; (2) SRIT at 4 months; and (3) TAU at 4 months.

There was a clear difference in age between SRIT and TAU at baseline, and age was found to be an important predictor for PAM and RAS. Therefore, a complementary mixed model analysis was carried out where age was added as a covariate. Complementary analysis was also done for other baseline characteristics (diagnosis etc.) but these gave the same results and are not shown.

The confidence level was set to 95 % and a *p*-value ≤0.05 was considered statistically significant. No interim analysis was conducted. Item level missing data were managed according to the rules of the specific questionnaires. The statistical analyses were carried out using IBM SPSS, version 22.0 [39] and R, version 2.13.1 [40].

## Results

**Fig. 1:**
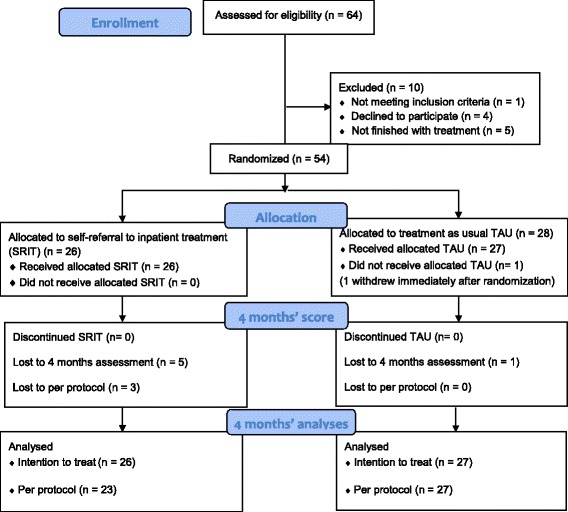
Study flow diagram

The flow of participants through the trial is illustrated in Fig. 1. Sixty-four participants were evaluated for eligibility. Ten had not completed their inpatient treatment, did not meet the inclusion criteria or did not want to participate in the study. One patient in the TAU group withdrew from the study directly after randomization. The final sample thus consisted of 53 participants with 26 patients in the SRIT group who received a SRIT contract. The control group consisted of 27 patients who were put on a 1 year waiting list before they got a contract on SRIT.

Five patients in the SRIT group and one in the TAU group did not complete questionnaire at 4 months (as they were not able to attend the 4 months assessment).

The total sample existed of 22 women and 31 men. Nearly three out of four had a diagnosis of schizophrenia. The rest had bipolar disorders, and 24 % of all patients in addition had abuse disorders. Patient characteristics were similar in both groups at baseline, except for age (Table 1). Age was different in the two groups, with the average age of SRIT participants being 10 years older than that of the TAU group. However, age was not related to follow-up measurements when controlling for baseline measurement.

**Table 1:** Patient characteristics at baseline for SRIT and TAU. Numbers are n (%) unless otherwise specified

	*SRIT* *n = 26*	*TAU* *n = 27*
Age, Mean (SD)	45.7 (12.6)	35.2 (11.7)
Gender		
Woman	12 (46.1)	10 (37.0)
Men	14 (53.9)	17 (63.0)
Diagnosis (ICD 10)		
Schizophrenia and bipolar disorders ^a^		
Schizophrenia and other psychotic disorders	18 (69.2)	22 (81.5)
Bipolar disorders	8 (30.8)	5 (18.5)
Comorbid diagnosis	10 (38.4)	7 (26.0)
Substance disorder	8 (30.8)	5 (18.5)
Living situation		
Living in relation with other	4 (15.4)	3 (11.1)
Living alone	22 (84.6)	24 (88.9)
Life income		
Work	1 (3.8)	1 (3.7)
Sickness benefits/courses /disability/retirement	22 (84.6)	22 (81.5)
Student	3 (11.5)	4 (14.8)
Received services from Psychiatric Ambulatory Rehabilitation Team	8 (30.8)	10 (37.0)

*SRIT* Self-referral to inpatient treatment

*TAU* Treatment as usual

^a^ As several patients had more than one diagnosis, the sum is larger than 100 %

### Implementation of intervention

Participants having an inpatient stay at the CMHC when they were randomized should be discharged within a day or two. This turned out to not be the case for many of the participants and this influenced the number of inpatient days, but there was no significant difference between the groups. Thirty-three (62.3 %) participants (18 in SRIT and 15 in TAU) were recruited while they were staying at the CMHC, and they stayed for in average another 13.6 days, median (interquartile range) was 4 (0–15.5), range 115 days before discharge; SRIT 10.8 days, median 5 (0–18), range 48 and TAU 16.4 days, median 2 (0–14), range 115. These inpatient days were counted as one admission during this 4 months’ intervention period. Eight (30.8 %) of 26 participants in SRIT and 12 (44.4 %) of 27 in TAU were discharged the same day or not staying at the CMHC when being randomized.

During the 4 months, 15 (57.7 %) of the 26 participants in the SRIT group utilized self-referral to inpatient treatment. In ‘number of admissions’, the mean was 0.8, the median was 1 (0–1) range 3 and the mean in ‘inpatient days’ was 2.9 and the median 1.5 (interquartile range 0–5) range 12. The 15 who had used the self-referral had on average 1.4 SRIT admissions (median 1 (1–2) range 2) and stayed on average 5 days (median 5 (3–7) range 11). In addition, this group had on average 2.8 (median 2 (2–4) range 5) admissions to the CMHC and hospital with on average 25.1 (median 18 (8–32) range 88) number of days. Two of the participants were transferred to an ordinary stay at the CMHC directly after 5 days SRIT use, and a third to the acute ward.

There were 11 persons (42 %) in the SRIT group who did not have a SRIT stay and nine of these (82 %) had other inpatient admissions, i.e. they used only ordinary admissions in the study period. Those nine had 1.2 admissions (median 1 (1–2) range 3), and 24.6 days (median 12, (3–33) range116) at the CMHC. Combining hospital and CMHC they had 1.6 admissions (median 1 (1–3) range 4) and 29.2 days (median 19, (3–38) range 117).

In the SRIT group 76.9 % (mean 1.1, SD = 0.9) of the participants were ordinary admitted to the CMHC and 74.1 % (mean 1.0, SD = 0.8) in the TAU group. The mean number of inpatient days was 20.3 (SD = 28.1) in SRIT and 23.7 (SD = 35.0) in TAU.

In total 24 (92 %) participants in SRIT were admitted through SRIT and ordinary admissions both at the CMHC and the psychiatric hospital ward. The number of mean admissions was 2.3 and median was 2 (1–4) range 6. In the control group 20 (74.1 %) of the participants were admitted to the CMHC and the psychiatric hospital ward. The mean in number of admissions was 1.3, and the median was 1 (0–2) range 4.

The mean in number of inpatient days (including both the CMHC and psychiatric hospital ward) in SRIT was 26.9 and the median was 18.5 (8–34) range 117. In the control group the number of inpatient days was 24.8 and the median 6 (0–39) range 119.

There was no significant difference between the groups in total inpatient days (*p* = 0.132). The SRIT group had replaced 10.8 % of their inpatient days with inpatient days due to self-referral. There was a significant difference (*p* = 0.01) in the total number of admissions (SRIT, ordinary stay at CMHC and psychiatric hospital ward) where the SRIT group had more admissions than the TAU group. More details are given in Table 2.

**Table 2:** Type of mental and somatic health care used from baseline to 4 months. Mean (SD, standard deviation) and median (IQR, interquartile range)

Mental and somatic health care	Intervention (SRIT)		Treatment as usual (TAU)		*P*-value ^a^
	(*N* = 26)		(*N* = 27)		
	Mean (SD)	Median (IQR)	Mean (SD)	Median (IQR)	
Admissions					
Total number	2.3 (1.5)	2 (1–4)	1.3 (1.2)	1 (0–2)	0.009
Self referral (SRIT)	0.8 (0.9)	1 (0–1)			
Community mental health center (CMHC)	1.1 (0.9)	1 (0.75–2)	1.0 (0.8)	1 (0–2)	
Psychiatric Hospital	0.4 (0.7)	0 (0–1)	0.3 (0.5)	0 (0–0)	
Inpatient days					
Total number	26.9 (28.1)	18.5 (8–34)	25.0 (35.3)	6 (0–39)	0.132
SRIT	2.9 (3.4)	1.5 (0–5)			
CMHC	20.3 (28.9)	11.5 (1.5–26.3)	23.7 (35.0)	5 (0–34)	
Psychiatric Hospital	3.7 (9.8)	0 (0–1)	1.0 (2.4)	0 (0–0)	
Outpatients visits					
Total number	12.7 (11.9)	8.5 (3–24.7)	17.7 (15.9)	14 (2–31)	0.388
Mental health outpatient	4.4 (4.4)	3.5 (1–8)	6.1 (8.6)	2 (0–13)	
Psychiatric Ambulatory Rehabilitation Team	5.7 (10.8)	0 (0–3)	9.9 (13.9)	0 (0–23)	
Supervised physical training ^b^	1.7 (6.3)	0 (0–0)	1.0 (5.0)	0 (0–0)	
Somatic outpatient	0.8 (2.2)	0 (0–0)	0.6 (1.3)	0 (0–1)	

^a^ Mann-Whitney *U* test. Only calculated for total number

^b^ Supervised physical training for persons with mental health problems with the possibility for short individual feedback on current health situation

### Outcomes

The intention to treat between-group analyses showed no significant differences after 4 months on patient activation (95 % confidence interval [CI] = −5.5 to 10.8, *p* = 0.52,) or recovery (95 % CI = −0.3 to 0.3, *p* = 0.92) (Table 3).

**Table 3:** Observed mean at baseline and 4 months, and mixed model based ITT analyses at 4 months for change within groups from baseline to 4 months and difference between groups at 4 months

	Observed				Estimated (mixed model)				
					Estimated value	Within Groups		Between Groups	
	Baseline		4 months		4 months	Baseline to 4 months		4 months	
	Mean (SD)	*n*	Mean (SD)	*n*	Mean (95 % CI)	Change (95 % CI)	*p*	Diff (95 % CI)	*p*
PAM									
All	64.0 (59.7, 68.3)	53	62.6 (57.8, 67.3)	47				2.7 (-5.49 to 10.79)	0.523
SRIT	64.1 (57.6, 70.6)	26	64.1 (58.1, 70.1)	21	64.7 (58.26 to 71.04)	0.6 (-5.84 to 7.12)	0.846		
TAU	63.9 (57.8, 69.9)	27	61.3 (53.9, 68.7)	26	62.0 (56.19 to 67.79)	-2.0 (-7.92 to 3.90)	0.505		
RAS									
All	3.7 (3.5, 3.9)	53	3.8 (3.6, 4.0)	47				0.01 (-0.27 to 0.30)	0.920
SRIT	3.8 (3.5, 4.1)	26	3.9 (3.6, 4.2)	21	3.9 (3.61 to 4.12)	0.2 (-0.04 to 0.39)	0.119		
TAU	3.6 (3.3, 3.9)	27	3.7 (3.4, 4.0)	26	3.9 (3.61 to 4.08)	0.2 (-0.04 to 0.35)	0.115		

*PAM* Patient Activation Measure (range from 0–100, an increase in scores indicates improvement)

*RAS* Recovery Assessment Scale (range from 1 to 5 in scores indicate improvement)

*SRIT* Self-referral to inpatient treatment (intervention group)

*TAU* Treatment as usual (control group)

The within-group comparisons showed no significant changes from baseline to 4 months on either patient activation or recovery in either of the two groups (Table 3).

There emerged no significant differences between the groups in scores on PAM (*p* = 0.91) or RAS (*p* = 0.86) when age was included as a covariate in the mixed model.

## Discussion

The Self-referral to inpatient treatment contract was used by 58 % of the SRIT participants and SRIT counted for 11 % of all inpatient days and 35 % of the admissions for the intervention group. This did not lead to any changes in patient activation and recovery. Our hypothesis was thus rejected.

### Implementation of intervention

Patients with a SRIT contract had a significantly higher total number of inpatient admissions due to their SRIT admissions. The increase in referrals is in line with previous studies on self-referral to inpatient treatment [12, 21, 41], which indicates that the SRIT at least in the beginning is an add on. In one study the admissions frequency increased with 158 % during 1 year of intervention [21] and in another study by 52 % during 9 months intervention [41]. This might not be a bad thing from a patient perspective as SRIT gives them the opportunity to regulate admissions according to their own perceived need. Even small changes in signs and symptoms can predict future disorders up to 10 weeks in advance [10], thus it might be beneficial with more admissions in the long run.

The finding that the increased number of admissions did not lead to increased number of inpatient days supports a cautious approach of viewing increased admissions as a problem. It indicates that service users with a SRIT contract prefer more frequent but shorter stays. However, previous observational studies on self-referral to inpatient treatment have found a decrease in the number of inpatient days [12, 21, 41].

There could be several reasons for why 9 (35 %) of all the SRIT participants had only used ordinary inpatient stay without using SRIT. The SRIT intervention was new both for the participants and the staff. Even if the staff and the participants were given verbal and written information about the study before and during the study period, they could have followed traditions and used ordinary referrals during the intervention period. One example is referral for “time outs” (e.g. during Christmas) which intendedly the self-referral could be used for. One could also expect that the participants had less need for inpatient treatment in the 4 months follow up period, as they might be in a more stable mental period due to more than half of the participants being discharged when they were included. However, there were only two participants with a SRIT contract and seven participants in the control group who did not have an inpatient stay during the intervention period, meaning that this is not a likely explanation. Another explanation could be that a SRIT contract might be seen as treatment in itself, a satisfaction and guarantee for an available place when needed, and not only connected to experiences of active use [12, 13]. This could explain some of the low use of SRIT. However, as only two participants were not admitted to the services during the intervention period and 35 % of the all SRIT participants had traditional stay at the CMHC instead, this explanation seems either not to be valid.

The 14 days’ quarantine time between each stay could have affected the use of SRIT, and may have increased the use of ordinary referrals for some of the service users. Two of the SRIT patients wanted to stay longer than 5 days and were transferred to ordinary stay instead of using SRIT. A need for longer stay than 5 days could be one explanation using ordinary stay instead of SRIT. The quarantine time itself may seem to be strict and differing to the philosophy behind the self-referral, and we support the recommendation to avoid a quarantine period in future projects [12].

The stay in CMHC (SRIT and ordinary admission) in both groups could be due to the familiarity to the staff. Both groups were carefully taken care of by the interviewers, staff and by their therapists and received a high level of care whether they received SRIT or TAU. SRIT participants were encouraged to make contact if they needed, while TAU participants might receive TAU after applying for admission. These factors could have contributed to increased admission in the groups. In addition, those in TAU were offered a SRIT contract after 1 year if they fulfilled the criteria at that point. Our impression was that most of them were highly motivated to behave in a manner that qualified them to get a SRIT.

### Patient activation and recovery

There was no effect of SRIT in patient activation after 4 months. The levels of patient activation both at baseline and at 4 months was better than found in a sample of new referrals patients waiting for outpatient treatment [29]. The participants in the present study had used mental health services for at least 2 years and had learned how to get in touch with their contacts in the unit at the CMHC and in the hospital. The fact that many of the participants were inpatients or in a discharge phase, and thereby potentially in a more mentally stable condition could have affected the level of patient activation. The time between baseline measures and follow-up was relatively short, and may have been too short to disclose the full effect of the SRIT contract on patient activation.

There was no effect of SRIT in recovery. This is not in line with and somewhat in contrast to what we found in a qualitative study which was nested within the present RCT [42]. Before analyzing the result of this RCT, what the patients in the intervention group said about how they coped 4 months after signing the contract for self-referral, was compared with the patients in the control group. It was found that patients with a contract for self-referral had more confidence in strategies to cope with mental disorders and to use more active cognitive strategies [42]. This is in line with another qualitative study showing that self-referral improves the service user’s confidence and ability to cope [13]. In the nested qualitative study, it was also found that patients with a contract of SRIT expressed less resignation, hopelessness and powerlessness than patients without a contract [42]. Regaining authority through interventions supporting empowerment is important for re-establish and preserve hope of recovery [43]. SRIT may thus be experienced as having important personal significance in terms of self-determination, but this was not evident in the recovery outcome measure used in this study. Other outcome instruments measuring self-efficacy, motivation and empowerment might be more appropriate than the measurements used in this randomized controlled trial.

The stability in patient activation and recovery scores in the intervention group might confirm, together with the results from the qualitative studies [13, 42], that giving patients with severe mental health diagnoses the opportunity to self-refer to inpatient treatment is safe and does not lead to increased use of health services. Replacing ordinary admissions with self-referral reduces the use of medical doctors and other resources to accomplish the referral process, and might reduce health service costs. This effect should be investigated further.

### Strengths and limitations

The present study is the first RCT measuring the effects of a SRIT contract. Furthermore, the use of well-known and valid questionnaires (PAM and RAS) strengthens the study.

Nonetheless, certain limitations should be taken into account when interpreting the results. Participants were not systematically diagnosed using the standardized diagnostic instruments [44], but were diagnosed according to ICD-10 following clinical observations across at least 2 years. This might have led to some error in diagnoses. Another limitation is that many patients were still admitted when they were randomized, and they could not start the intervention as expected. The number of participants could be higher and given a better basis for generalizing the effect of SRIT.

Before each scoring the participants were informed that the questionnaires referred to mental health, but there was no guarantee that this was properly considered when responding. We have focused on general patient activation (PAM) and recovery. This may not be sufficient to reveal subtler, but important changes following a self-referral period. It may also be that our chosen population did not include the type of patients who can benefit the most from self-referral.

## Conclusion

The study revealed no significant effects from a self-referral contract on patient activation and recovery. Giving persons with severe mental disorders the possibility to self-refer to inpatient treatment did not lead to significantly increased use of health services. Replacing ordinary in-patient treatment with self-referral should be investigated with respect to cost-effectiveness. Further studies are required to draw firm conclusions on the long-term effects from self-referral to inpatient treatment in mental health care.

## Abbreviations

PAM: Patient activation measure
RAS: Recovery assessment scale
SRIT: Self-referral to inpatient treatment
TAU: Treatment as usual

## References

[1] Thornicroft G, Alem A, Antunes Dos Santos R, Barley E, Drake RE, Gregorio G, Hanlon C, Ito H, Latimer E, Law A (2010). WPA guidance on steps, obstacles and mistakes to avoid in the implementation of community mental health care. World Psychiatry.

[2] Hamann J, Cohen R, Leucht S, Busch R, Kissling W (2005). Do patients with schizophrenia wish to be involved in decisions about their medical treatment?. Am J Psychiatry.

[3] Farrelly S, Brown G, Rose D, Doherty E, Henderson RC, Birchwood M, Marshall M, Waheed W, Szmukler G, Thornicroft G (2014). What service users with psychotic disorders want in a mental health crisis or relapse: thematic analysis of joint crisis plans. Soc Psychiatry Psychiatric Epidemiol.

[4] Gudde CB, Olso TM, Antonsen DO, Ro M, Eriksen L, Vatne S (2013). Experiences and preferences of users with major mental disorders regarding helpful care in situations of mental crisis. Scand J Public Health.

[5] Kukla M, Salyers MP, Lysaker PH (2013). Levels of patient activation among adults with schizophrenia: associations with hope, symptoms, medication adherence, and recovery attitudes. J Nerv Ment Dis.

[6] Storm M, Edwards A (2013). Models of user involvement in the mental health context: intentions and implementation challenges. Psychiatr Q.

[7] Newman D, O’Reilly P, Lee SH, Kennedy C (2015). Mental health service users’ experiences of mental health care: an integrative literature review. J Psychiatr Ment Health Nurs.

[8] Slade M, Amering M, Farkas M, Hamilton B, O’Hagan M, Panther G, Perkins R, Shepherd G, Tse S, Whitley R (2014). Uses and abuses of recovery: implementing recovery-oriented practices in mental health systems. World Psychiatry.

[9] Thornicroft G, Tansella M (2013). The balanced care model for global mental health. Psychol Med.

[10] Morriss R, Vinjamuri I, Faizal MA, Bolton CA, McCarthy JP. Training to recognise the early signs of recurrence in schizophrenia (Review). Cochrane Database Syst Rev. 2013;(2):CD005147. doi:10.1002/14651858.CD005147.pub2. 10.1002/14651858.CD005147.pub2 PMC12066188 23450559

[11] Biringer E, Sundfør B, Davidson L, Hartveit M, Borg M. Life on a waiting list: How do people experience and cope with delayed access to a community mental health center? Scandinavian Psychologist. 2015;2:e6. http://psykologisk.no/sp/2015/04/e6/.

[12] Strand M, von Hausswolff-Juhlin Y (2015). Patient-controlled hospital admission in psychiatry: A systematic review. Nord J Psychiatry.

[13] Olso TM, Gudde CB, Moljord IE, Evensen GH, Antonsen DO, Eriksen L (2016). More than just a bed: mental health service users’ experiences of self-referral admission. Int J Ment Health Syst.

[14] Green CA, Perrin NA, Polen MR, Leo MC, Hibbard JH, Tusler M (2010). Development of the Patient Activation Measure for mental health. Adm Policy Mental Health.

[15] Hibbard J, Gilburt H: Supporting people to manage their health. An introduction to patient activation. In: The King’s fund Ideas that change health care. Accuracy Matters edn. London: The King’s Fund; 2014.

[16] Salyers MP, Matthias MS, Spann CL, Lydick JM, Rollins AL, Frankel RM (2009). The role of patient activation in psychiatric visits. Psychiatr Serv.

[17] Shepherd G, Boardman J, Slade M (2008). Making recovery a reality.

[18] Oxford Centre for Evidence-based Medicine – Levels of Evidence (March 2009). http://www.cebm.net/oxford-centre-evidence-based-medicine-levels-evidence-march-2009/.

[19] Health and Social Affairs: Veileder til forskrift om individuell plan. Guide to regulations on individual plans. Edited by The Norwegian Directorate of Health. Oslo; 2005.

[20] Ministry of Health and Care Services: Veileder om rehabilitering, habilitering, individuell plan og koordinator. Guide to rehabilitation, habilitation, individual plan and coordinator. Edited by The Norwegian Directorate of Health. Oslo; 2015.

[21] Heskestad S, Tytlandsvik M (2008). Patient-guided admissions for severe psychotic conditions. Tidsskr Nor Laegeforen.

[22] Hibbard JH, Mahoney ER, Stockard J, Tusler M (2005). Development and testing of a short form of the patient activation measure. Health Serv Res.

[23] Hibbard JH, Stockard J, Mahoney ER, Tusler M (2004). Development of the Patient Activation Measure (PAM): conceptualizing and measuring activation in patients and consumers. Health Serv Res.

[24] Insignia Health: Patient Activation. http://www.insigniahealth.com/.

[25] Zill JM, Dwinger S, Kriston L, Rohenkohl A, Harter M, Dirmaier J (2013). Psychometric evaluation of the German version of the patient activation measure (PAM13). BMC Public Health.

[26] Skolasky RL, Green AF, Scharfstein D, Boult C, Reider L, Wegener ST (2011). Psychometric properties of the patient activation measure among multimorbid older adults. Health Serv Res.

[27] Packer TL, Kephart G, Ghahari S, Audulv A, Versnel J, Warner G (2015). The Patient Activation Measure: a validation study in a neurological population. Qual Life Res.

[28] Steinsbekk A (2008). Patient Activation Measure. Tidsskr Nor Laegeforen.

[29] Moljord IE, Lara-Cabrera ML, Perestelo-Perez L, Rivero-Santana A, Eriksen L, Linaker OM (2015). Psychometric properties of the Patient Activation Measure-13 among out-patients waiting for mental health treatment: A validation study in Norway. Patient Educ Couns.

[30] Corrigan PW, Salzer M, Ralph RO, Sangster Y, Keck L (2004). Examining the factor structure of the recovery assessment scale. Schizophr Bull.

[31] Cavelti M, Kvrgic S, Beck EM, Kossowsky J, Vauth R (2012). Assessing recovery from schizophrenia as an individual process. A review of self-report instruments. Eur Psychiatry.

[32] Corrigan PW, Giffort D, Rashid F, Leary M, Okeke I (1999). Recovery as a psychological construct. Community Ment Health J.

[33] Burgess P, Pirkis J, Coombs T, Rosen A (2011). Assessing the value of existing recovery measures for routine use in Australian mental health services. Austr N Z J Psychiatry.

[34] Chiba R, Miyamoto Y, Kawakami N (2010). Reliability and validity of the Japanese version of the Recovery Assessment Scale (RAS) for people with chronic mental illness: scale development. Int J Nursing Stud.

[35] Salzer MS, Brusilovskiy E (2014). Advancing recovery science: reliability and validity properties of the Recovery Assessment Scale. Psychiatr Serv.

[36] Rosenthal R, Rosnow RL (2008). Essentials of behavioral research: Methods and data analysis.

[37] Chan A, Tetzlaff JM, Gøtzsche PC, Altman DG, Mann H, Berlin JA (2013). SPIRIT 2013 explanation and elaboration: guidance for protocols of clinical trials. BMJ.

[38] Twisk J, de Boer M, de Vente W, Heymans M (2013). Multiple imputation of missing values was not necessary before performing a longitudinal mixed-model analysis. J Clin Epidemiol.

[39] Corp IBM (2013). IBM SPSS Statistics for Windows, Verson 22.0.

[40] R Development Core Team. R: A Language and Environment for Statistical Computing. Vienna: R Foundation for Statistical Computing; 2011. ISBN: 3-900051-07-0. http://www.R-project.org/.

[41] Støvind H, Hanneborg EM, Ruud T (2012). Bedre tid med brukerstyrte innleggelser? (‘Better time with user-controlled admissions’). Sykepleien.

[42] Rise MB, Evensen GH, Moljord IE, Ro M, Bjorgen D, Eriksen L (2014). How do patients with severe mental diagnosis cope in everyday life - a qualitative study comparing patients’ experiences of self-referral inpatient treatment with treatment as usual?. BMC Health Serv Res.

[43] Samuelsen SS, Moljord, IEO, Eriksen L: Re-establishing and preserving hope of recovery through user participation in patients with a severe mantal disorder: the self-referral-to-inpatient-treatment project. Nurcing Open 2016, 1-5. doi:10.100/nop2.59. 10.1002/nop2.59 PMC5050546 27708833

[44] First MB, Spitzer RL, Gibbon M, Williams JB (eds.): Strucutured Clinical Interview for DSM-IV-TR Axis I Disorders, Research Version, Patient Edition. (SCID-I/P). New York State Institute: Biometric Research; 2002.

